# Modelling changes in glutathione homeostasis as a function of quinone redox metabolism

**DOI:** 10.1038/s41598-019-42799-2

**Published:** 2019-04-19

**Authors:** Ross A. Kelly, Joseph Leedale, Dominic Calleja, Steven J. Enoch, Andy Harrell, Amy E. Chadwick, Steven Webb

**Affiliations:** 1 Department of Applied Mathematics, Liverpool John Moores University, Byrom Street, Liverpool, L3 3AF UK; 2 EPSRC Liverpool Centre for Mathematics in Healthcare, Department of Mathematical Sciences, University of Liverpool, Liverpool, L69 7ZL UK; 3 Institute for Risk and Uncertainty, University of Liverpool, Liverpool, L69 7ZF UK; 4 School of Pharmacy and Biomolecular Sciences, Liverpool John Moores University, Byrom Street, Liverpool, L3 3AF UK; 5 GlaxoSmithKline, David Jack Centre for Research, Park Road, Ware, SG12 0DP UK; 6 MRC Centre for Drug Safety Science, Department of Molecular and Clinical Pharmacology, University of Liverpool, Ashton Street, Liverpool, L69 3GE UK

**Keywords:** Biochemical reaction networks, Bioenergetics

## Abstract

Redox cycling is an understated mechanism of toxicity associated with a plethora of xenobiotics, responsible for preventing the effective treatment of serious conditions such as malaria and cardiomyopathy. Quinone compounds are notorious redox cyclers, present in drugs such as doxorubicin, which is used to treat a host of human cancers. However, the therapeutic index of doxorubicin is undermined by dose-dependent cardiotoxicity, which may be a function of futile redox cycling. In this study, a doxorubicin-specific *in silico* quinone redox metabolism model is described. Doxorubicin-GSH adduct formation kinetics are thermodynamically estimated from its reduction potential, while the remainder of the model is parameterised using oxygen consumption rate data, indicative of hydroquinone auto-oxidation. The model is then combined with a comprehensive glutathione metabolism model, facilitating the simulation of quinone redox cycling, and adduct-induced GSH depletion. Simulations suggest that glutathione pools are most sensitive to exposure duration at pharmacologically and supra-pharmacologically relevant doxorubicin concentrations. The model provides an alternative method of investigating and quantifying redox cycling induced oxidative stress, circumventing the experimental difficulties of measuring and tracking radical species. This *in silico* framework provides a platform from which GSH depletion can be explored as a function of a compound’s physicochemical properties.

## Introduction

Redox cycling describes the continuous reduction and oxidation cycle of a compound, forming radical intermediates capable of transferring an electron to molecular oxygen, generating the superoxide radical anion (O_2_^.−^)^1^. The dismutation of O_2_^.−^ results in the formation of hydrogen peroxide (H_2_O_2_), which can lead to elevated levels of other potentially harmful reactive oxygen and nitrogen species (ROS, RNS)^2^. Quinone species are arguably the most renowned redox cyclers and are pivotal to many biological mechanisms^3^. For example, quinone-based redox cycling facilitates electron transport within the mitochondria via ubiquinone (Coenzyme Q), and is therefore essential to cellular bioenergetics^4^. However, futile quinone redox cycling can lead to a cascade of ROS formation and as such, the link between toxicity and quinone redox cycling is widely acknowledged^5^. Nevertheless, redox cycling remains an understated mechanism of toxicity due to the fleeting existence of free radical intermediates which hinders their quantification in real time, both *in vitro* and *in vivo*^6^. Potential redox cycling-based toxicity has been implicated with many compounds^7^. One such quinone containing compound, doxorubicin, is an anthracycline drug used to treat a variety of human cancers and is regarded as one of the most important chemotherapeutic agents^8^. However, the therapeutic utility of doxorubicin is undermined by its dose-dependent cardiotoxicity, possibly arising as a result of futile redox cycling, with NADH dehydrogenase within the mitochondria proposed as the most likely site of anthracycline reduction^9,10^.

**Figure 1:**
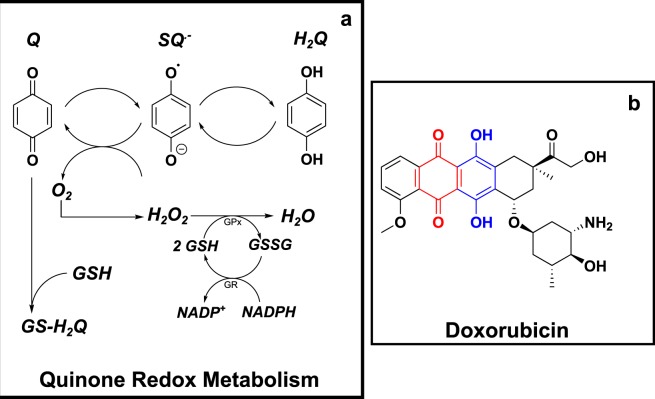
(**a**) Quinone redox cycling, ROS formation and GSH-based detoxification. A schematic of the single electron reduction of a quinone (*Q*) to a semiquinone radical anion (*SQ*^.−^), followed by complete reduction to the hydroquinone (*H*_*2*_*Q*). The figure shows the concomitant reduction of molecular oxygen by *SQ*^.−^ to form the ROS, superoxide (*O*_*2*_^.−^), followed by its dismutation into hydrogen peroxide (*H*_*2*_*O*_*2*_), which is detoxified by glutathione (*GSH*) into harmless H_2_O through the glutathione peroxidase (GPx) reaction. GSH is regenerated from its oxidised form (*GSSG*), catalysed by the glutathione reduction (GR) reaction. Finally, the glutathione-quinone adduct (GS-H_2_Q) formation represents the reductive addition (Michael reaction) between GSH and the Q electrophile. (**b**) Chemical structure of doxorubicin. The anthracycline contains both the quinone (red) and hydroquinone (blue) moieties within its chemical structure. The hydroquinone is the site of auto-oxidation for doxorubicin.

Cellular glutathione plays a major role in the defence against redox cycling-derived oxidative stress, either by direct interaction with ROS, RNS and electrophiles, or by acting as a co-factor for various enzymes^11,12^. As a result, glutathione is at the forefront of mitigating quinone-derived toxicity, by enzymatically reducing redox-generated H_2_O_2_ to harmless H_2_O via glutathione peroxidase, or by direct reaction and detoxification of the quinone electrophile (Fig. 1)^13–15^.

The ability of a quinone to redox cycle is dependent upon the favourability of its reduction by a single electron, which can be quantitatively described by its reduction potential (*E*^*o*^). *E*^*o*^ is the standard reduction potential in volts (V), when measured under standard conditions: 25 °C, 1.0 M, pH 0.0 when in aqueous solution and at a pressure of 100 kPa (0.986 atm)^16^. The reduction potential can be described at non-standard conditions (i.e. physiological conditions) using the Nernst Equation (Eq. 1), where *E* is the reduction potential at non-standard conditions, *E*^*o*^′ is the standard reduction potential at pH 7.0, *R* is the universal gas constant (8.3145 J mol^−1^ K^−1^), *T* is the temperature of interest in Kelvin, *F* is the Faraday constant (9.6485 × 10^4^ C mol^−1^), *n* is the number of electrons in the reduction reaction and *Q*_*r*_ is the reaction quotient for the half-cell reaction; equal to the ratio of the reduced and oxidised species:

1 $$E={E}^{o^{\prime} }-\frac{RT}{nF}\,\mathrm{ln}\,{Q}_{r}.$$

The susceptibility of molecular oxygen to be reduced to O_2_^.−^ may also be described by its reduction potential, shown in Equation (2). Specifically, the ability of a semiquinone radical anion (SQ^.−^) to reduce molecular oxygen into superoxide can be quantified by linking both reduction potentials^16^.

2 $$S{Q}^{.-}+{O}_{2}\leftrightharpoons Q+{O}_{2}^{.-}$$

The thermodynamic favourability of the reaction between SQ^.−^ and molecular oxygen can therefore be assessed by considering *E*^*o*^′ for the (Q/SQ^.^) and (O_2_/O_2_^.−^), where *E*^*o*^′ (O_2_/O_2_^.−^) is −180 mV^17^. The reaction equilibrium constant *K*_*eq*_ can also be calculated using Equation (3):

3 $${E}^{{o}^{{\rm{^{\prime} }}}}(\frac{{O}_{2}}{{{O}_{2}}^{.-}})-{E}^{{o}^{{\rm{^{\prime} }}}}(\frac{Q}{S{Q}^{.-}})=\frac{RT}{F}\,{\rm{l}}{\rm{n}}\,{K}_{eq}.$$

According to Equation (3), if *E*^*o*^′ (Q/SQ^.−^) is less than *E*^*o*^′ (O_2_/O_2_^.−^), then the equilibrium will favour O_2_^.−^ formation (Equation (2). Similarly, if *E*^*o*^′ (Q/SQ^.−^) is greater than *E*^*o*^′ (O_2_/O_2_^.−^) then the opposite is true, favouring the reverse reaction, thereby leaving superoxide formation thermodynamically unfavourable. However, it is important to recognise that these reactions are reversible and therefore superoxide formation can occur even if the reverse rate is greater than the forward rate. Within the cell, the production of superoxide is then a function of other biological factors that influence the position of the equilibrium, such as, for example, detoxification by superoxide dismutase enzymes (SOD)^3,18^.

The reductive addition reaction between Q and GSH is also linked to reduction potential (*E*^*o*^′), whereby the second order rate constant, dependent on the concentration of the electrophile and GSH, (log(k/M^−1^ s^−1^)) for the Michael reaction may be estimated^3^. This is extremely useful, as it provides a kinetic handle on the non-redox cycling quinone-derived GSH depletion. Ultimately, toxicity is likely to present when the cellular antioxidant defense mechanisms, such as glutathione metabolism, are overwhelmed. Indeed, depletion of cellular GSH by 20–30% of normal has been shown to result in impaired oxidative stress defence and lead to dell death^19^.

In this study, an *in silico* model of quinone metabolism was used to investigate how redox cycling-induced ROS production and reductive addition GSH adduct formation impacts glutathione homeostasis. This work aims to determine the significance of both quinone-based GSH depletion mechanisms, as well as proposing a mathematical framework that can help circumvent the experimental difficulty of quantifying reactive radical intermediates in real time. Using doxorubicin as a training compound, three models of quinone redox cycling were constructed, each capturing different potential redox cycling mechanisms. Each model was fitted to oxygen consumption rate data, indicative of ROS formation, allowing the estimation of unknown kinetic parameters, with the best fit model selected using variance-based sensitivity analysis, the Akaike Information Criterion (AIC) and Bayesian Information Criterion (BIC). The selected model was then expanded to include GS-H_2_Q adduct formation, estimating the required kinetic formation parameter from its reduction potential, *E*^*o*^′, before being combined with a previously published model of glutathione metabolism. The combined model was used to simulate the subsequent GSH depletion as a function ROS generated from redox cycling and adduct formation for a single and extended exposure of doxorubicin.

## Materials and Methods

### In silico

#### Model construction

**Figure 2:**
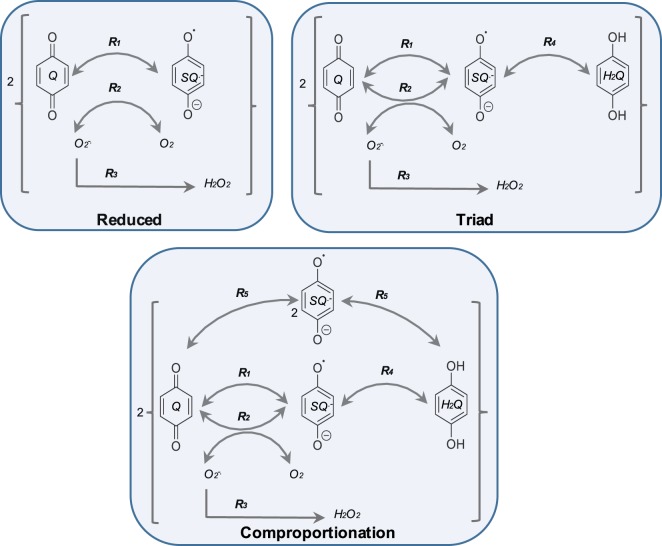
Doxorubicin-quinone redox cycling model schematics. Three variations of quinone redox cycling (reduced, triad and comproportionation) are described. Each model comprises of a single compartment and a selection of the following species: quinone (*Q*); semiquinone radical (*SQ*^.−^); hydroquinone (*H*_*2*_*Q*); superoxide radical (*O*_*2*_^.−^); molecular oxygen (*O*_*2*_); and hydrogen peroxide (*H*_*2*_*O*_*2*_). The corresponding reaction rate equations (*R*_*1*–*5*_) are described in Table 1.

Three *in silico* quinone redox metabolism models were proposed, each representing different potential redox cycling mechanisms (Fig. 2). The first model, hereafter referred to as the reduced model, is a reduced representation describing the cycling between the parent quinone (Q) and semiquinone radical anion (SQ^.−^), as well as superoxide (O_2_^.−^) and hydrogen peroxide (H_2_O_2_) formation. The second model (triad model) represents the classical triad of quinone redox cycling, expanding the reduced model to include the transformation between the semiquinone radical anion and fully reduced hydroquinone. The third model (comproportionation model) expands the triad model to include the comproportionation reaction, whereby two semiquinone radicals can reform the parent quinone compound and hydroquinone species. A schematic of each model is shown in Fig. 2, with the corresponding kinetic rate equations described in Table 1.

**Table 1:** Model kinetic expressions.

Reaction	Rate Equation
*R*_*1*_	${R}_{1}={k}_{p1}\,[Q]-{k}_{m1}\,[SQ],$
*R*_*2*_	${R}_{2}={k}_{p2}\,[SQ]\,{k}_{O2}-{k}_{m2}\,[Q]\,[{O}_{2}^{.-}],$
*R*_*3*_	${R}_{3}={k}_{p3}\,{[SQ]}^{2},$
*R*_*4*_	${R}_{4}={k}_{p4}\,[SQ]-{k}_{m4}\,[{H}_{2}Q],$
*R*_*5*_	${R}_{5}={k}_{p5}\,{[SQ]}^{2}-{k}_{m5}\,[Q]\,[{H}_{2}Q].$

Kinetic terms assembled to describe quinone/doxorubicin redox cycling are based on the law of mass action. All parameters values were obtained from fitting to experimental data and are located in the supplementary information. All reactions correspond to Fig. 2 only.

#### Model kinetic terms, parameters and initial conditions

Quinone redox reaction kinetics were described according to the law of mass action such that the rate of reaction is proportional to the concentration of the reactants for a given a rate constant, with *k*_*pn*_ and *k*_*mn*_ representing the forward rate and reverse rate constants respectively and *n* denotes the considered reaction (1–5; Table 1). Initial-conditions, fitted parameter values and ordinary differential equations (ODEs) are provided in the supplementary information. The model ODEs were solved in MATLAB^®^ 2017a via numerical integration using the variable-order stiff solver ode15s.

#### Model selection: AIC-BIC criteria

Model selection was directed using the Akaike Information Criterion (AIC) and Bayesian Information Criterion (BIC). AIC and BIC values examine how fitted model solutions compare to the experimental OCR data. Both AIC and BIC values are penalised-likelihood criterion that consider model complexity (e.g. number of parameter values) and are commonly used during model selection^20^. For example, AIC and BIC values may suggest that a less complex model (fewer parameters) may be the most appropriate model to use, even if a more complex model fits the data better.

#### GSH metabolism expansion

Expansion of the selected model to include adduct formation (Equation (4)) was achieved by estimating the reaction rate constant, *k*_*QGSH*_, from Fig. 12 in the work by Song. *et al*.^3^, using the doxorubicin-specific *E*^*o*^′ (−292 to −341 mV). This particular figure demonstrates how the rate constants for the Michael addition of glutathione with various quinones are a function of the *E*^*o*^′ with a linear relationship.

4 $$Q+GSH\mathop{\to }\limits^{{k}_{QGSH}}GS\text{-}{H}_{2}Q.$$

Simulating the effects of redox cycling and adduct formation on GSH homeostasis was achieved by extending the triad quinone metabolism model to include a complete representation of glutathione metabolism. A full curated version of the GSH metabolism model developed by Reed *et al*.^21^ was downloaded from the BioModels Database and amended for simulation and coupling to the quinone metabolism model in MATLAB. Specifically, the Reed model and quinone redox cycling models were coupled via the H_2_O_2_, Q and GSH variables. Model H_2_O_2_ is generated from quinone redox cycling/hydroquinone auto-oxidation, being detoxified by glutathione peroxidase, and Q and GSH removal is a function of adduct formation (Michael reaction). The amended ODEs for H_2_O_2_, GSH and Q are as follows:

$$\begin{array}{rcl}\frac{d[{H}_{2}{O}_{2}]}{dt} & = & {R}_{3}-{V}_{GPx}+{R}_{6},\\ \frac{d[GSH]}{dt} & = & {V}_{GS}-{V}_{cgshHb}-{V}_{cgshLb}-2\,{V}_{GPx}+2\,{V}_{GR}-{V}_{gsh{\deg }}-{R}_{5},\\ \frac{d[Q]}{dt} & = & -\,{R}_{1}+{R}_{2}-{R}_{5},\end{array}$$

where, $${R}_{5}=[Q]\,[GSH]\,{k}_{QGSH}$$ and $${R}_{6}={[{O}_{2}^{.-}]}^{2}{k}_{SOD}$$.

The rate equations for *V*_*Gs*_, *V*_*cgshHb*_, *V*_*cgshLb*_, *V*_*GPx*_, *V*_*GR*_ and *V*_*gshdeg*_ are taken from Reed *et al*.^21^ and located within the supplementary information, along with the full details of their model. The successful combination of this model with the redox cycling model was proven by recapitulation of Figs 2 and 6 from the Reed *et al*. publication^21^, shown in the supplementary information.

#### Quinone redox metabolism model assumptions

Like all mathematical models, this biochemical redox cycling model is based upon a set of assumptions regarding biological and chemical space. The model assumes that the electrons and factors mediating reductive processes, such as reductase enzymes, are abundant and that doxorubicin reduction occurs readily. Furthermore, the concomitant rate of auto-oxidation of doxorubicin within the cell is assumed to be equal to that of the OCR experimental data used to parameterise the model.

## Experimental

### Materials

All extracellular flux analysis consumables were purchased from Agilent (North Billerica, MA, USA). Doxorubicin was purchased from Sigma Aldrich (Dorset, UK).

### Extracellular flux analysis (EFA)

The utility plate was calibrated according to manufacturer instructions on the day before the assay. Doxorubicin stock solution (10 mM, 100% DMSO) was serially diluted in unbuffered seahorse assay medium to prepare 6 concentrations: 400, 300, 200, 100, 80 and 40 µM. Compound solutions were set to a final pH of 7.0 using HCl and KOH when necessary. Final compound dilution occurs post-injection giving a final concentration of 50, 35, 25, 12.5, 10 and 5 µM inside the well.

On the day of the assay, doxorubicin working solutions were added to injection port A of each well of the sensor cartridge (25 µL). The instrument was then calibrated according to the manufacturer’s instructions. Prior to analysis, the XFe96 instrument (Seahorse Biosciences, North Billerica, MA, USA) mixed the assay media in each well for 10 minutes to allow the oxygen partial pressure to reach equilibrium. Extracellular flux analysis was conducted simultaneously measuring the extracellular acidification rate ECAR via proton production rate (PPR) and oxygen consumption rate (OCR). The first three measurements were used to establish a baseline rate. All measurements include a 3-minute mix, allowing the probe to retract and collapse the transient micro chamber. This allows oxygen tension and pH in the microenvironment to restore to normal. Doxorubicin was injected after the third measurement (16 minutes) and the resulting changes in PPR and OCR were measured for a further 20 measurements (over 150 minutes) yielding the basal response.

## Results

### Experimental

#### Extracellular flux analysis: oxygen consumption rate (OCR)

**Figure 3:**
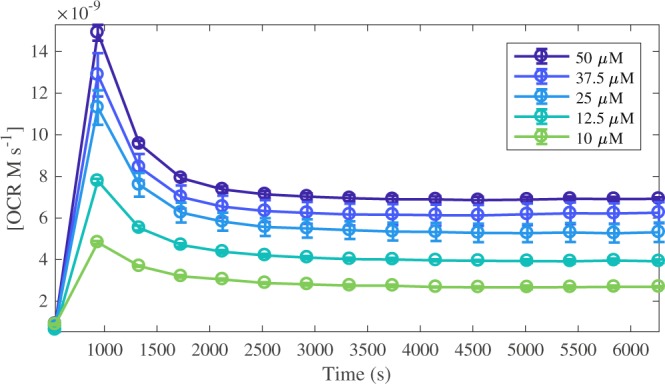
Oxygen consumption rate (OCR) profiles for doxorubicin at 50, 37.5, 25, 12.5 and 10 μM. Each data point in represents the OCR immediately after a 3-minute solution mix within the well, measured in the transient microchamber. Compound injection occurs at t = 16 min (between measurements 1 and 2). Each dataset is the average of n = 3 experiments expressed with its standard deviation.

Extracellular flux analysis was used to measure the oxygen consumption rate before and after injection of doxorubicin into unbuffered XF media at pH 7.0 (Fig. 3). Doxorubicin (50, 37.5, 25, 12.5 and 10 μM) was injected into the media 16 minutes into the experiment (between measurements 1 and 2), yielding concentration-dependent oxygen consumption profiles in a cell-free environment. Oxygen consumption has long been attributed to hydroquinone auto-oxidation and the formation of H_2_O_2_ and O_2_^.−^ ^3,5,22^. Auto-oxidation refers to oxidation in the absence of a metal catalyst and in this instance, the oxygen consumption rate data represents doxorubicin hydroquinone (Fig. 1b) auto-oxidation^23^. Hydroquinone auto-oxidation yields stoichiometric production of H_2_O_2_, shown in Equation (5).

5 $${H}_{2}Q+{O}_{2}\mathop{\to }\limits^{k}{H}_{2}{O}_{2}+Q$$

Note, Equation (5) is not intended to describe the complete oxidation mechanisms by which hydroquinone auto-oxidates to generate H_2_O_2_. The actual mechanism is likely to occur via two sequential steps with semiquinone (SQ^.−^) and superoxide (O2^.−^) intermediates. Rather, it aims to provide concise stoichiometric representation of the formation of H_2_O_2_ from H_2_Q auto-oxidation.

### In silico

#### Model fitting and selection: AIC BIC Criterion

The oxygen consumption rate (OCR) data generated in Fig. 3 provides an experimental platform for the parameterisation of the mathematical models. The OCR data indirectly corresponds to H_2_O_2_ production, via the superoxide formation reaction shown in Equation (6). Within the models, the dynamics of O_2_ are not explicitly specified but rather, assumed to be constant as *k*_*Q*2_, due to a separation of scales. Consequently, OCR is then represented by the *R*_*2*_ reaction flux, as shown in Equation (6).

6 $$OCR={R}_{2}={k}_{p2}[S{Q}^{.-}]{k}_{O2}-{k}_{m2}\,[Q]\,[{O}_{2}^{.-}].$$

The reduced, triad and comproportionation models were fit to the 37.5 and 25 μM (training concentrations) OCR data profiles from Fig. 3 via the *R*_*2*_ reaction flux shown in Equation (6). The 50, 12.5 and 10 μM data (test concentrations) were withheld to be used for blind validation. The unknown rate constants for *R*_*1*_, *R*_*2*_, *R*_*3*,_ *R*_*4*_ and *R*_*5*_ for the respective models were fitted using the LSQNONLIN function in MATLAB, a non-linear least squares solver. The performance of each model was compared by examining how close the predicted solutions were to the experimental OCR data using the penalised-likelihood AIC and BIC criteria. AIC and BIC values are representative of the distance between the fitted likelihood of the model and the unknown true likelihood function of the data, with the BIC criterion penalising model complexity more heavily than the AIC^20,24^. Table 2 shows the computed AIC and BIC values, illustrating that the reduced model returns a much higher score than the triad and comproportionation models. However, the AIC and BIC values for the triad and comproportionation models are very similar. Therefore, this criterion was deemed conclusive enough to discount the reduced model as a viable model for describing the OCR data, but insufficient to prompt selection of either the triad or comproportionation model.

**Table 2:** AIC and BIC values for the reduced, triad and comproportionation model fits of the OCR data.

	Reduced Model	Triad Model	Comproportionation Model
*AIC*	3.878 × 10^4^	3.239 × 10^3^	3.244 × 10^3^
*BIC*	3.879 × 10^4^	3.247 × 10^3^	3.253 × 10^3^

#### Sensitivity analysis: Model selection

**Figure 4:**
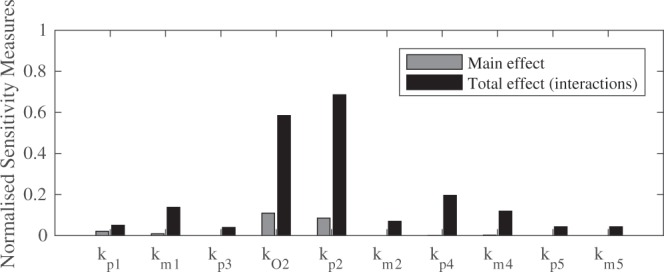
Normalised sensitivity measures for the comproportionation model reaction rate constants, expressed as main and total effects.

Global sensitivity analysis (GSA) was conducted using the classical Sobol method. This variance-based method is concerned with the decomposition of the output variance and attributing this variance to input factors^25,26^. In this instance, GSA was performed to quantify the influence of all model parameters on model OCR output (Fig. 4), facilitating selection of either the triad or comproportionation model.

The results of the global sensitivity analysis for the redox cycling parameters with respect to model OCR output are shown in Fig. 4. Normal distributions were applied to all inputs, with the mean obtained from the previous optimisation procedure, and coefficient of variation of 180%. First order main effect indices and the total-order indices were computed. The main effect indices are equivalent to direct variance-based measures; they measure the effect of varying an input factor alone, averaged over variations in all other inputs. The total effect indices provide the contribution of variance in a parameter including the variance of all possible higher order interactions, indicating the importance of any input. The use of total effect indices negates the need for determining higher order interactions which can be computationally expensive. The sensitivity of the forward rate constants for the superoxide formation reaction, *k*_*p2*_, and the oxygen concentration parameter, *k*_*O2*_, proved to be the most sensitive globally with respect to total effects, highlighted in Fig. 4. Furthermore, both *k*_*p2*_ and *k*_*O2*_ register as the two most sensitive parameters with respect to the main effects, with *k*_*p4*_ also sensitive. The sensitivity of *R*_*4*_ parameters (*k*_*p4*_) with respect to model OCR output is in good accordance with the AIC/BIC analysis, confirming the need for the *R*_*4*_ reaction, which is present in both the triad and the comproportionation models, but not in the reduced model. This suggests that the reformation of the SQ^.−^ from H_2_Q is mechanistically important during the redox metabolism of doxorubicin.

The global sensitivity analysis suggests that the comproportionation reaction, *R*_*5*_, is not pivotal for the model OCR output, given that its parameters, *k*_*p5*_ and *k*_*m5*_, are the two least sensitive parameters for both main and total interactions. This finding prompted the selection of the triad model, given that first; *R*_*4*_ is required to adequately model OCR output, illustrated in both the AIC/BIC analysis and GSA, second; that model OCR output is insensitive to the inclusion of the comproportionation kinetics (*R*_*5*_) and finally; the triad model is simpler, requiring estimation of fewer parameters.

#### Triad model validation

**Figure 5:**
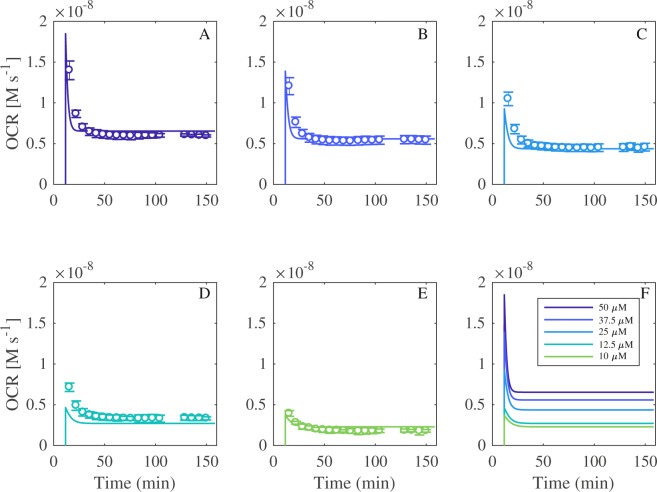
Triad model fitting and simulation. Comparison of simulated and experimental OCR data for 50, 37.5, 25, 12.5 and 10 µM of doxorubicin (Fig. 3).

Figure 5 compares the simulated triad model OCR output with the experimental OCR data. The model was first fitted to the “training concentrations” (37.5 and 25 µM doxorubicin), and then used to simulate the “test concentrations” (50, 12.5 and 10 µM doxorubicin) as a means of blind validation, showing good accordance between the experimental and *in silico* outputs for all concentrations of doxorubicin. The simulated profiles for the reduced and comproportionation models, as well as the parameter values generated from the fitting process, are located in the supplementary information.

#### Combined triad-GSH metabolism model simulations: quinone metabolism and ROS production

**Figure 6:**
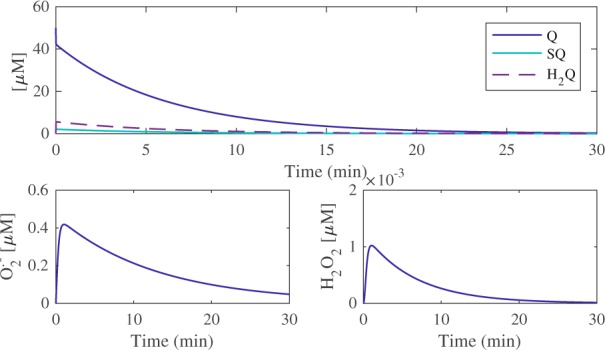
Model simulations for doxorubicin and ROS metabolism. The fate of a single doxorubicin exposure (50 µM) was simulated over a 30-minute time-span in order to glean the resulting transformations between Q, SQ^.−^ and H_2_Q (top panel). The resulting superoxide and hydrogen peroxide formation and detoxification profiles are illustrated in the bottom left and right panels, respectively.

Following the parameterisation and sensitivity analysis, the triad model was expanded to include GS-H_2_Q adduct formation (quinone removal) and was combined with the glutathione metabolism model constructed by Reed *et al*.^21^. The combined model output was validated by replicating Figs 2 and 6 from the Reed *et al*. publication (shown in the supplementary information) in order to confirm that; i), the model obtained from the BioModels Database can recapitulate the figures that were not used for its curation, and ii) that addition of the triad model does not affect the glutathione metabolism model output when no doxorubicin is present.

A major benefit of implementing an *in silico* approach to investigating quinone metabolism is the ability to simulate and visualise radical species, such as superoxide, in real time, as this is essentially inaccessible experimentally both *in vitro* and *in vivo*. Figure 6 presents the simulated fate of a single 50 μM doxorubicin exposure (top panel), as well as the subsequent O_2_^.−^ and H_2_O_2_ profiles, over a 30-minute time-span. The model predicts that a single 50 μM exposure will yield a rapid but small increase in H_2_Q and SQ^.−^ of 5.57 μM and 2.10 μM respectively, with all three forms of the quinone requiring 30 minutes to be removed by GSH from the system.

#### Combined triad-GSH metabolism model simulations: impact of quinone metabolism on glutathione homeostasis

**Figure 7:**
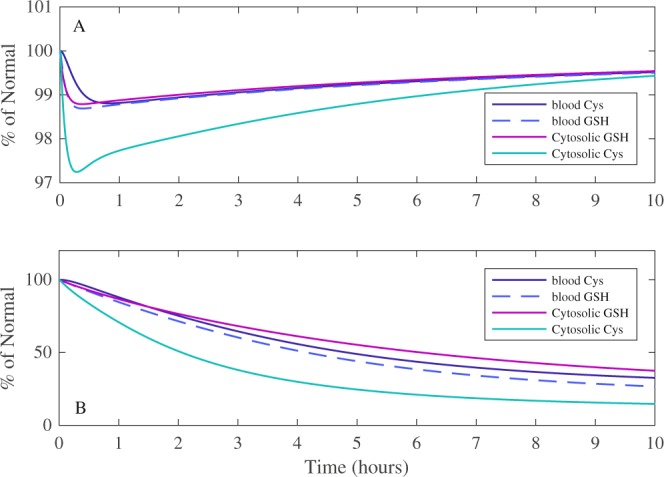
The effects of doxorubicin quinone-based metabolism on glutathione and cysteine model homeostasis. The resulting simulated changes in blood and cytosolic GSH and cysteine after a single or constant exposure to 50 μM of doxorubicin are shown in (**A** and **B**) respectively, for a 10-hour time-span. % of normal represents the percentage difference of the variable compared to its simulated steady state value.

The model was used to investigate how single *vs* constant 50 μM exposure of doxorubicin influenced blood and cytosolic GSH and cysteine (Cys) levels, over a 10-hour time-span, shown in Fig. 7. A single exposure (A) causes a small decrease (less than 3% of normal), in both blood and cytosolic GSH and Cys concentrations, with levels returning to above 99% of normal for all species within the simulated time-span. Cytosolic Cys experiences the greatest decrease after a single exposure, which is indicative of the model facilitating rapid GSH re-synthesis after an initial depletion. While cytosolic and blood GSH and Cys biochemical species all experience depletion, the model predicts that a single exposure to doxorubicin only results in a minimal perturbation of the antioxidant defence system. In contrast, a constant 50 μM exposure of quinone (B) overwhelms blood and cytosolic GSH and Cys, showing no signs of recovery after a 10-hour time-span. Specifically, simulations suggest that blood Cys and GSH are reduced by 68.7% and 74.1% respectively, whereas cytosolic Cys and GSH are reduced by 81.2% and 64.6% respectively. While a constant exposure of quinone is not necessarily representative of an *in vivo* scenario, it is however, much more representative of an *in vitro* situation, whereby a constant source of quinone is essentially available in the extracellular media during cell culture^27^. Therefore, the model provides a platform from which the impact of varying degrees of quinone concentration and exposure times on GSH homeostasis may be computationally examined.

A practical application of the combined model would be to predict the concentration and exposure time required to cause toxicity via overwhelming GSH metabolism as a function of quinone metabolism. As such, the model was used to simulate how long it would take pharmacologically and toxicologically relevant concentrations of doxorubicin to yield a 70% reduction in GSH concentration, indicative of impaired cellular antioxidant defence, protein binding and cell death^19^.

**Figure 8:**
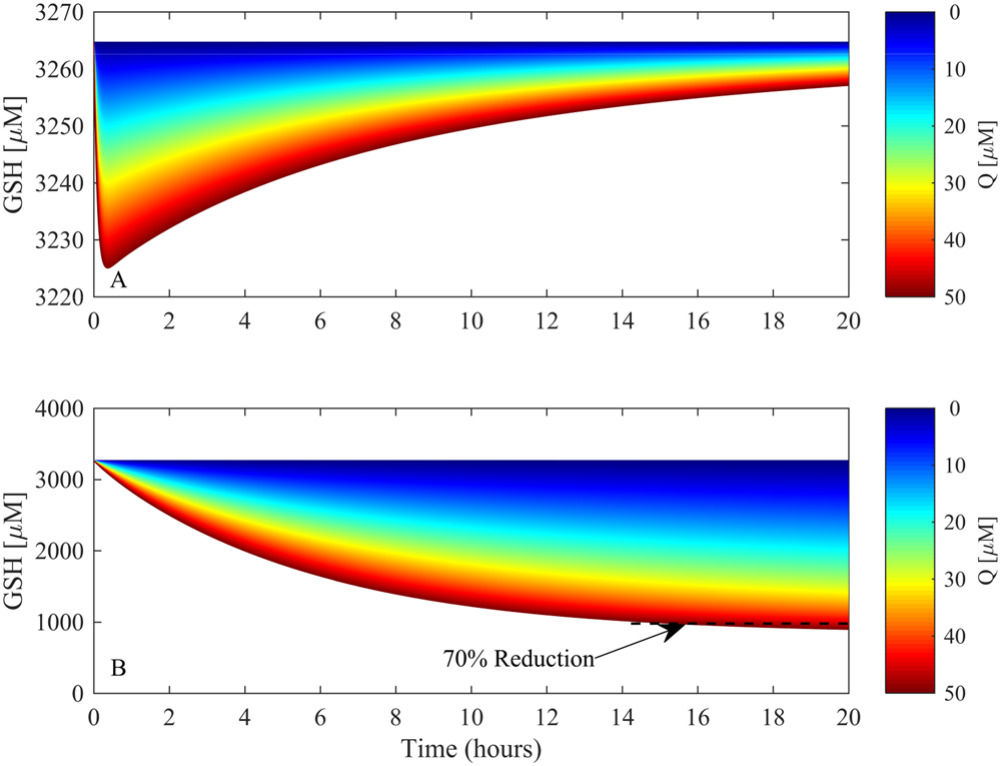
The effects of doxorubicin quinone-based metabolism on glutathione homeostasis. The resulting simulated changes in cytosolic GSH following single (**A**) or constant exposure (**B**) to a range of doxorubicin concentrations (0–50 μM) are shown in (**A** and **B**) respectively, for a 20-hour time-span. The 70% reduction threshold is indicated in (**B**) with a black dashed line.

Figure 8 illustrates how a single exposure *vs* constant infusion of a wide range of doxorubicin concentrations (0–50 μM) affects cytosolic GSH concentration. The simulation time-span was extended to 20 hours in order to discern what exposure time and concentration would yield a 70% of normal reduction of GSH. A single exposure of doxorubicin between 0 and 50 μM, yields minimal cytosolic GSH depletion (2.5% maximum decrease), which recovers within the prescribed time-span (Fig. 8A). However, for the same concentration range, a constant exposure can yield a harmful depletion of GSH to 30% of normal after 14 hours, indicated by the black-dashed line (Fig. 8B).

## Discussion

A mathematical modelling approach was deployed to simulate GSH depletion as a function of doxorubicin redox metabolism. The predominant motivation for mathematically modelling quinone redox metabolism was to facilitate the investigation of experimentally difficult scenarios; specifically, the fleeting existence of radical species and the rapid rate at which redox cycling can generate ROS, causing oxidative stress^5,7,28^. Doxorubicin was selected as a training compound for three reasons: i) the quinone moiety, present in doxorubicin, is acknowledged as a notorious redox cycler; ii) doxorubicin also contains the hydroquinone moiety as part of its chemical structure, allowing auto-oxidation to be used as a parameterisation method for a potential redox cycle; and iii) doxorubicin itself has long been implicated with futile redox cycling toxicity, specifically within the mitochondria^29^.

Mathematically modelling redox cycling is difficult because of the different potential mechanisms through which the process may occur. However, these difficulties were reduced by first considering three models (Fig. 2), with increasing degrees of mechanistic complexity, to be aligned with the experimental data. This method allowed the goodness of fit to inform upon an appropriate model structure when the actual reaction rates were unknown and, in doing so, provided a useful indication of the possible mechanism by which this process occurs. Appropriate model assumptions facilitated initial model reduction by capturing only the essential elements of the system. In this study, it was assumed that using the auto-reduction of the hydroquinone adjacent to the quinone on doxorubicin was a sufficient indicator of the rate of oxidation of a potential redox cycle, either on the hydroquinone or the quinone after reduction. Indeed, experimental analysis showed that introduction of doxorubicin into physiologically relevant pH media yielded a concentration-dependent oxygen consumption profile, revealing free (non-metal catalysed) auto-oxidation (Fig. 3). This finding was in good accordance with the literature, where the hydroquinone is routinely shown to auto-oxidate under these conditions^30,31^.

AIC and BIC model selection criteria were deployed to suggest which model is mechanistically important, guided by the OCR data. The analysis revealed that both the comproportionation and triad models were better suited to represent the experimental data than the reduced model, but were inconclusive with respect to overall model selection, given that both AIC/BIC values were extremely similar (Fig. 4). The triad model was selected as the final model as the comproportionation reaction (*R*_*5*_) parameters (*k*_*p5*_ and *k*_*m5*_) were the two least sensitive parameters with respect to model OCR output for both main and total effects during global sensitivity analysis (Fig. 4). While the comproportionation reaction is a well reported redox cycling chemical mechanism, in this instance the triad model does not require the additional comproportionation reaction to accurately replicate the experimental OCR profile, suggesting that the comproportionation reaction is not mechanistically integral to the production of ROS through a quinone-based redox cycle. Omission of the comproportionation kinetics by selecting the triad model also reduces the number of parameters that require estimating, reducing uncertainty in the overall parameter space.

Global sensitivity analysis revealed that *k*_*02*_ (oxygen concentration) is the most sensitive parameter for OCR and ROS output, suggesting that biological environments with elevated oxygen presence could be more susceptible to redox-induced ROS. Indeed, the association of doxorubicin with bioenergetic toxicity is well stated in the literature^32–34^. The global sensitivity analysis also confirmed the importance of the redox cycle between SQ^.−^ and H_2_Q (*R*_*4*_), showing that the reverse rate constant, *k*_*m4*_ (SQ^.−^ reformation), was the second most sensitive (main interactions) with respect to model OCR and therefore ROS production, suggesting auto-oxidation is an essential part of the doxorubicin redox mechanism. The triad model captures the OCR data for all concentrations of doxorubicin, simulating auto-oxidation via the formation of SQ^.−^ and O_2_^.−^ intermediates, which is in good accordance with the literature^3^. While the simulations are confined by these assumptions, it is worth noting that redox cycling is governed by thermodynamics, which according to the reduction potential of doxorubicin, are favourable with respect to the formation of superoxide should a semiquinone radical species be present^3^.

After the model was expanded to include GS-H_2_Q adduct formation as a function of its reduction potential, it was then combined with the Reed glutathione model^21^ in order to simulate how the quinone redox-metabolism of doxorubicin influenced the glutathione metabolism. The combined model presented here effectively extends the work of Reed *et al*. to investigate how quinone redox metabolism can cause toxicity through GSH depletion. The Reed model provides a comprehensive mathematical representation of one-carbon GSH metabolism, boasting the inclusion of the transsulfuration pathway, as well as glutathione synthesis, transport and breakdown. Model curation provides researchers with the ability to obtain, adapt and implement such mathematical models, as outlined in this study, and is therefore a powerful tool in the arsenal of any systems biologist, pharmacologist or toxicologist. The combined model first provided visualisation of quinone redox-metabolism by capturing the transitions between Q, SQ^.−^ and H_2_Q, as well as the subsequent production of O_2_^.−^ over a 30-minute time-span. Note that semiquinone radicals can possess extremely long half-lives, up to days at 37 °C^35^, and as such, it is unsurprising that SQ^.−^ is present over 30 minutes in the model (Fig. 6). The simulations suggested that quinone metabolism yielded an increase in ROS (O_2_^.−^ and H_2_O_2_), producing a maximum of 0.42 μM and 1 × 10^−3^ μM respectively. Interestingly, despite the presence of superoxide dismutase (SOD) (*k* = 2.4 × 10^9^ M^−1^ s^−1^)^36^, the resulting concentration of H_2_O_2_ is significantly smaller than the concentration of superoxide, indicating that the model is able to respond well to a transient increase in ROS, maintaining low H_2_O_2_ concentrations. It is important to note that current *in vitro* redox cycling detection is centred on indirect quantification of H_2_O_2_ production and O_2_ consumption rather than direct measurement of dynamic radical species over time^22,28^.

The model predicted that for 10-hour-long toxicologically relevant doxorubicin (50 μM) simulations, the duration of the exposure is more important than concentration with respect to overwhelming glutathione metabolism. The consideration of cysteine during these simulations were important, as cytosolic cysteine is the rate limiting amino acid precursor for synthesis of GSH, via the γ-glutamylcysteine synthetase (GCS) enzyme, and this is a function of its reduced concentration compared to the other precursors, glycine and glutamate. Consequently, cysteine availability and the resulting GCS activity are both pivotal for GSH re-synthesis and therefore provide an indication of the model’s potential to recover GSH levels^21^.

Further simulations showed that over a wide range of doxorubicin (0–50 μM), the model can be used to suggest the specific concentration and exposure duration required to deplete cytosolic GSH by 70%, the threshold by which antioxidant defence is impaired, protein binding occurs, and cell death is possible. A broad range was considered in order to explore the supra-pharmacological (>10 μM) concentrations required to induce toxicity, as well as the effects of more pharmacologically relevant values (0.1–1.0 μM) for an extended duration^10^. The influence doxorubicin has on GSH depletion is most certainly also dependent upon the cell-type and tissue-type in question. For example, lung cancer cell-lines show different sensitivities to doxorubicin in the form of GSH depletion, with A549 and GLC_4_210(S) cells experiencing approximately 50% and 64% GSH depletion after a 12 hour exposure to 70 nM and 5 nM (per million cells) respectively^37^. However, HeLa cells are much more sensitive, with 2.5 nM (per million cells) of doxorubicin resulting in up to 80% GSH depletion for the same exposure time^38^. Some cell-types are much less sensitive to doxorubicin-induced GSH depletion. Hepatocytes treated with 111 μM for 4 hours experience an approximate 20% decrease in both cytoplasmic and mitochondrial GSH^39^. Our model simulations are reflective of the hepatic GSH environment, agreeing with the supra-pharmacological concentrations of doxorubicin required to illicit comparable GSH depletion in the liver^39^. Indeed, the original GSH metabolism constructed by Reed *et al*. explores the properties of glutathione metabolism in the liver^21^, therefore lending confidence to our predictions. Training a mathematical model to other specific cell-lines is possible and beneficial to reveal phenotypic heterogeneity in metabolic properties. Such methodology has been successfully applied^40^, and could be implemented in this framework to investigate specific cell and tissue types.

The modelling approach we have utilised in this study facilitates the exploration of potential toxicity based on a compound’s physicochemical properties, in this instance the reduction potential. The ability to predict compound concentrations and exposure durations that could cause a significant compromise in cellular antioxidant defence as a function of a physicochemical property, especially with respect to an understated mechanism such as redox cycling, could prove to be extremely useful when investigating toxicity with the reduction of animal models in mind. In this instance, the concentrations of doxorubicin required to induce a deleterious GSH response fall firmly outside of the therapeutic ranges of circulating doxorubicin reported^40^. Consequently, simulations suggest that while doxorubicin redox metabolism impacts GSH metabolism, the concentrations required to illicit a toxic response, with either a single or extended exposure, reside outside that of the therapeutic dosing range. This finding agrees with the literature whereby the role of quinone redox metabolism is an ambiguous source of toxicity, with evidence suggesting that redox cycling requires supra-pharmacological concentrations of doxorubicin to generate substantial ROS in tissues and cells^10^.

Overall, the combined model demonstrates the utility of high quality previously published models when constructing a framework to investigate a specific toxicity. The combined quinone redox – glutathione metabolism model can be used to simulate experimentally challenging scenarios such as potential redox cycling toxicity, while providing a platform from which quinone exposure and concentration toxicity experiments may be guided. Furthermore, the construction of mathematical frameworks such as this can be implemented to explore other classes of compounds and mechanisms of toxicity as a function of their physicochemical properties, while providing an alternative method of quantifying experimentally elusive radical species.

## Supplementary information

Supplementary information

Dataset 1

## Data Availability

All model parameters and kinetic information are presented in the supplementary information. Experimental oxygen consumption rate data is provided as an additional file.
