# Supplementary material for: Modelling changes in glutathione homeostasis as a function of quinone redox metabolism
Source: Sci Rep. 2019 Apr 19;9:6333. doi: 10.1038/s41598-019-42799-2 (PMC6474874; doi:10.1038/s41598-019-42799-2)
Supplement: Supplementary file 1 — Supplementary information [file 41598_2019_42799_MOESM1_ESM.pdf]

SUPPLEMENTARY INFORMATION

Modelling the changes in glutathione homeostasis as a function of quinone redox metabolism

Ross A Kelly[1], Joseph Leedale[2*], Dominic Calleja[3], Steven J Enoch[4], Andy Harrell[5], Amy E Chadwick[6] and Steven D Webb[1]

*Correspondence:
J.Leedale@liverpool.ac.uk
[2]EPSRC Liverpool Centre for Mathematics in Healthcare, Department of Mathematical Sciences, University of Liverpool, Liverpool, UK
Full list of author information is available at the end of the article

1 Summary

In this supplementary information, a complete description of the construction of the combined quinone redox-glutathione metabolism model is presented. Appendix A outlines the notation used to describe the model in terms of its variables and reactions. Appendix B describes the model kinetic equations. Appendix C presents the ordinary differential equations (ODEs). Appendix D illustrates the validation of the combined model, specifically, by recapitulating Figures 2 and 6 from the Reed. et. al study [1]. Appendix E illustrates the fitting to the oxygen consumption rate data for the reduced and comproportionation models. Appendix F describes the initial conditions for the quinone metabolism section of the model and points the reader to the BioModels Database for the glutathione metabolism initial conditions.

Appendix A: Notation

A.1 Enzyme and variable acronyms

A full list of variable and enzyme names present in the combined quinone-GSH metabolism model are described here. All glutathione metabolism variables and enzymes are taken from Reed. et. al. [1].

A.2 Non-enzyme reaction acronyms

A.3 Model variable names

Reed et. al. [1] use three prefixes, m, c and b, to differentiate between mitochondria, cytosol and blood compartments. Metabolites present in one compartment only, or metabolite concentrations that are assumed equal in different compartments have no prefixes.

A.4 Constant variables

Reed et. al. [1] assume that total cellular folate is divided equally between the cytosol and mitochondria, with the mitochondria occupying one quarter of the cell volume. In their study, folate is not transported into the cell nor is it catabolised. The total normal folate concentration is 13.3 μM in the cytosol and 40 μM.

Table 1 Enzyme names and acronyms

Abbreviation	Enzyme names
Mitochondrial folate cycle enzymes	
mSHMT	serinehydroxymethyltransferase
mFTD	10-formyltetrahydrofolate dehydrogenase
mFTS	10-formyltetrahydrofolate synthase
mFTS	10-formyltetrahydrofolate synthase
mMTCH	5,10-methenyltetrahydrofolate cyclohydrolase
mMTD	5,10-methylenetetrahydrofolate dehydrogenase
mNE	non-enzymatic conversion
DMGD	dimethylglycine dehydrogenase
SDH	sarcosine dehydrogenase
GDC	glycine decarboxylase (glycine cleavage system)
Cytosolic folate cycle enzymes	
cSHMT	serinehydroxymethyltransferase
DHFR	dihydrofolate reductase
cFTD	10-formyltetrahydrofolate dehydrogenase
cFTS	10-formyltetrahydrofolate synthase
cMTCH	5,10-methenyltetrahydrofolate cyclohydrolase
cMTD	5,10-methylenetetrahydrofolate dehydrogenase
MTHFR	5,10-methylenetetrahydrofolate reductase
AICART	aminoimidazolecarboxamide ribonucleotide transferase
PGT	Phosphoribosyl glycinamidetransformalase
cNE	non-enzymatic conversion
TS	thymidylate synthase
Methionine cycle enzymes	
MAT-I	methionine adenosyl transferase I
MAT-III	methionine adenosyl transferase III
GNMT	glycine N-methyltransferase
DNMT	DNA-methyltransferase
SAAH	S-adenosylhomocysteine hydrolase
MS	methionine synthase
BHMT	betaine-homocysteine methyltransferase
CBS	cystathionine β-synthase
glutathione metabolism enzymes	
CTGL	γ-cystathionase
GCS	γ-glutamylcysteine synthetase
GS	glutathione synthetase
GPX	glutathione peroxidase
GR	glutathione reductase

Table 2 Non-Enzymatic reaction names and acronyms

Quinone metabolism reactions	Description
R_1	Quinone single electron reduction (reversible)
R_2	Semiquinone oxidation - superoxide formation (reversible)
R_3	Superoxide dismutation
R_4	Semiquinone reduction (reversible)
R_5	Comproportionation
R_6	$GS - H_2Q$ adduct formation (Michael reaction)

Appendix B: Kinetics

B.1 Quinone redox metabolism kinetics

All quinone redox metabolism kinetics, including $GS - H_2Q$ adduct formation, are constructed via the law of mass action. Square brackets denote the concentration of a chemical species.

$$R_1 = k_{p1}[Q] - k_{m1}[SQ^{\cdot -}] \tag{1}$$

$$R_2 = k_{p2}k_{O_2}[SQ^{\cdot -}] - k_{m2}[Q][O_2^{\cdot -}] \tag{2}$$

$$R_3 = k_{p3}[O_2^{\cdot -}]^2 \tag{3}$$

$$R_4 = k_{p4}[SQ^{\cdot -}] - k_{m4}[Q] \tag{4}$$

Table 3 Variable names (μM)

Abbreviation	Variable names
Quinone redox metabolites and ROS	
Q	Quinone
$SQ^{\cdot -}$	Semiquinone radical anion
H_2Q	Hydroquinone (quinol)
H_2O_2	Hydrogen peroxide
$O_2^{\cdot -}$	Superoxide
mitochondrial folate cycle metabolites	
mthf	tetrahydrofolate
m2cf	5-10-methylenetetrahydrofolate
m1cf	5-10-methenyltetrahydrofolate
m10f	10-formyltetrahydrofolate
cytosolic folate cycle metabolites	
cthf	tetrahydrofolate
c2cf	5-10-methylenetetrahydrofolate
c1cf	5-10-methenyltetrahydrofolate
c10f	10-formyltetrahydrofolate
dhf	dihydrofolate
5mf	5-methyltetrahydrofolate
aic	P-ribosyl-5-amino-4-imidazole carboxamide
methionine cycle metabolites	
met	methionine
sam	S-adenosylmethionine
sah	S-adenosylhomocysteine
hcy	homocysteine
glutathione metabolites	
cyt	cystathionine
ccys	cytosolic cysteine
bcys	blood cysteine
glc	glutamyl-cysteine
cgsh	cytosolic glutathione
bgsh	blood glutathione
cgsg	cytosolic glutathione disulfide
bgsg	blood glutathione disulfide
other metabolites	
bgly	blood glycine
bglu	blood glutamate
mgly	mitochondrial glycine
mser	mitochondrial serine
mcoo	mitochondrial formate
Cglu	cytosolic glutamate
Cgly	cytosolic glycine
Cser	cytosolic serine
Ccoo	cytosolic formate
dmg	dimethylglycine
Src	sarcosine

Table 4 Constant variables

Abbreviation	Description	Concentration (μM)
GAR	glycinamide ribonucleotide	10
NADPH	nicotinamide adenine dinucleotide phosphate	50
BET	betaine	50
HCHO	formaldehyde	500
DUMP	deoxyuridine monophophate	20
Bser	blood serine	150
Bmet	blood methionine	30
FOL	total cellular folate	20

Michael-addition derived $GS - H_2Q$ adduct formation:

$$R_5 = k_{QGSH}[Q](gsh) \tag{5}$$

Superoxide dismutase:

$$R_6 = k_{SOD}[O_2^{\cdot -}]^2 \tag{6}$$

B.2 Reactions with standard kinetics

The glutathione metabolism model describes all reactions with standard enzyme kinetics using either the Michaelis-Menten term, or by a reversible bi-substrate random-order Michaelis-Menten term:

$$V = \frac{V_{max}[S]}{K_m + [S]} \tag{7}$$

$$V = \frac{V_{max}[S_1][S_2]}{(K_{m1} + [S_1])(K_{m2} + [S_2])} \tag{8}$$

The appropriate kinetic term is apparent when considering the corresponding parameter values in table 5.

Table 5 Kinetic parameters (time in hours, concentration in μM). All parameter values are taken from [1].

Parameter	Literature	Model	Parameter	Literature	Model
**AICART**			**CTGL**		
Km,10f	5.9-50	5.9	Km,cyt	500	500
Km,aic	10-100	100	Vmax	-	1500
Vmax	-	55000			
**DHFR**			**DMGD**		
Km,dhf	0.12-1.9	0.5	Km,dmg	50	50
Km,NADPH	0.3-5.6	4.0	Km,mthf	-	50
Vmax	350-23000	2000	Vmax	-	15000
**FTD**			**cFTS**		
Km,10f	0.9-20	20	Km,thf	0.1 - 600	3
cVmax	-	500	Km,coo	8 - 1000	43
mVmax	-	1050	Vmax	100 - 486000	3900
**mFTS**			**GDC**		
Km,thf	0.1 - 600	3	Km,mgly	3400-40000	3400
Km,coo	8 - 1000	43	Km,mthf	50	50 Vmax
100 - 486000	2000		Vmax	-	15000
Km,10f	-	22			
**GPX**			**GR**		
Km,gsh	1330	1330	Km,gsg	72, 107	107
Km,H2O2	-	.09	Km,NADPH	10.4	10.4
Vmax	-	4500	Vmax	-	8925
**MTCH**			**MTD**		
Km,1cf	4-250	250	Km	2-5	2
cVmax	880-1380000	500000	cVmax	520-594000	80000
mVmax	880-1380000	790000	mVmax	520-594000	180000
Km,10f	20-450	100	Km	1-10	10
Vmax	10.5-1380000	20000	Vmax	594000	600000
**PGT**			**SAHH**		
Km	.9-58	4.9	Km,sah	0.75-15.2	6.5
Km,GAR	520	520	Vmax	-	320
- Vmax	6600-16200	24300	Km,hcy	56.6-200	150
			Vmax	-	4530
**SHMT**			**SDH**		
Km,ser	350-1300	600	Km,mthf	-	50
Km,thf	45-300	50	Vmax	-	15000
cVmax	500-162000	5200			
mVmax	500-162000	11440			
Km,gly	3000-10000	10000			
Km	3200-10000	3200			
cVmax	12600-120000000	15000000			
mVmax	12600-120000000	30000000			
**TS**					
Km,DUMP	5-37	6.3			
Km	10-45	14			
Vmax	30-4200	5000			

B.3 Reactions with non-standard kinetics

Reed et. al. [1] provide a thorough description of the non-standard reaction kinetics in terms. As such, the reader is directed to their study for the derivation of these equations. This section will outline the kinetics and parameter values only.

B.3.1 BHMT

$$V = e^{.0021}(sam + sah)e^{.002(102.6)} \frac{V_{max}[hcy][BET]}{(K_{m1} + [hcy])(K_{m2} + [BET]} \cdot \left(\frac{H_2O_2 + K_a}{ssH_2O_2 + K_i} \right) \quad (9)$$

Parameter	Value
K_{m1}	12
K_{m2}	100
V_{max}	2160
ssH_2O_2	0.01
K_i	0.01

B.3.2 CBS

$$V = \left(\frac{V_{max}[hcy][_cser]}{(K_{m1} + [hcy])(K_{m2} + [_cser]} \right) \left(\frac{(1.086)(sam + sah^2)}{(30)^2 + (sam + sah)^2} \right) \left(\frac{H_2O_2 + K_a}{ssH_2O_{Ka}} \right) \quad (10)$$

Parameter	Value
K_{m1}	1000
K_{m2}	2000
V_{max}	420000
ssH_2O_2	0.035
K_a	0.01

B.3.3 DNMT

$$V = \frac{V_{max}(sam)}{K_m(1 + \frac{sah}{K_i} + sam)} \quad (11)$$

Parameter	Value
K_m	1.4
V_{max}	180
K_i	1.4

B.3.4 GCS

$$V = \frac{V_{max}((cys)(glu) - \frac{(glc)}{K_e}}{K_m^{cys}K_m^{glu} + K_m^{cys}(glu) + K_m^{glu}(cys)(1 + \frac{(gsh)}{k_i} + 1 + \frac{(glu)}{k_m^{glu}}) + \frac{(glu)}{K_m^{glu}} + \frac{(glc)}{K_p} + \frac{(gsh)}{K_i}} \cdot \left(\frac{ssH_2O_2 + K_a}{H2O2 + K_a}\right) \tag{12}$$

Parameter	Value
V_{max}	3600
K_e	5597
K_m^{cys}	100
K_m^{glu}	1900
K_i	8200
K_p	300
K_a	0.01
ssH_2O_22	0.01

B.3.5 GNMT

$$V = \left(\frac{V_{max}(sam)(gly)}{(K_{m1} + sam)(K_{m2} + gly)}\right)\left(\frac{1}{1 + \frac{sah}{K_i}}\right)\left(\frac{4.8}{0.35 + 5mf}\right) \tag{13}$$

Parameter	Value
V_{max}	260
K_{m1}	63
K_{m2}	130
K_i	18

GS

$$V = \frac{V_{max}((gsh)(glc) - \frac{(gsh)}{K_e})}{K_m^{glc}K_m^{gly} + (glc)K_m^{gly} + (gly)K_m^{glc}(1 + \frac{(glc)}{K_m^{glc}}) + \frac{(gsh)}{K_p}} \tag{14}$$

Parameter	Value
V_{max}	5400
K_e	5600
K_m^{gly}	300
K_m^{glc}	22
K_i	30

B.3.6 MAT-I

$$V = \left(\frac{V_{max}(met)}{K_m + met}\right)(0.23 + (0.8)e^{-0.0026(sam))}\left(\frac{K_i + 66.71}{K_i + gsg}\right) \quad (15)$$

Parameter	Value
V_{max}	260
K_i	2140
K_m	41

B.3.7 MAT-III

$$V = \left(\frac{V_{max}(met)^{1.21}}{K_m + (met)^{1.21}}\right)\left(1 + \frac{(7.2)(sam)^2}{(K_a)^2 + (sam)^2}\right)\left(\frac{K_i + 66.71}{K_i + gsg}\right) \quad (16)$$

Parameter	Value
V_{max}	220
K_i	4030
K_a	360
K_m	300

B.3.8 MS

$$V = \frac{V_{max}(5mf)(hcy)}{(K_m^{5mf} + 5mf)(K_m^{hcy} + hcy)}\left(\frac{ssH_2O_2 + K_i}{H_2O_2 + K_i}\right) \quad (17)$$

Parameter	Value
V_{max}	500
K_m^{5mf}	25
K_m^{hcy}	1
K_i	0.01
ssH_2O_2	0.01

B.3.9 MTHFH

$$V = \frac{V_{max}(2cf)(NADPH)}{(K_{m1} + 2cf)(K_{m2} + NADPH)} \cdot \frac{72}{10 + sam - sah} \quad (18)$$

Parameter	Value
V_{max}	5300
K_{m1}	50
K_{m2}	16

B.3.10 NE

$$V = k_1(thf)(HCHO) - k_2(2cf) \tag{19}$$

Parameter	Value
k_1	0.03
k_2 (Cytosol)	22
k_2 (Mitochondria)	20

B.4 Transport kinetics

The general formula for the kinetics of transport between the blood and the cytosol compartments take the following form:

$$V = \frac{V_{max}[bAA]}{K_m + [bAA]} - k_{out}[cAA] \tag{20}$$

where AA represents an amino acid and prefixes b and c refer to the blood and cytosolic compartments respectively. The formulation of the transport kinetics used in the glutathione metabolism model is explained in great detail in the Reed et. al manuscript [1]. Briefly, the transporter velocities from the blood to the cytosol, or mitochondria to the cytosol are denoted V_{bSERc} and V_{mSERc} respectively. The transporter units are μM/hr increase in the cytosol.

B.5 Quinone redox metabolism parameters

The quinone metabolism parameters were estimated using the LSQNONLIN function in MATLAB, by fitting the triad model to the experimental OCR data representive of hydroquinone auto-oxidation.

Appendix C: Model ordinary differential equations (ODEs)

The rate of each biochemical reaction is denoted with a V, representing a velocity of μMhr^{-1}. Model variables in different compartments are distinguished with subscripts (c, b, m) representing cytosolic, blood and mitochondrial variables.

$$\frac{d}{dt}\,{}_mthf = V_{mFTD} - V_{mSHMT} - V_{mFTS} - V_{mNE} - V_{GDC} - V_{SDH} - V_{DMGD} \tag{21}$$

Table 6 Amino acid transport parameters

Parameter	Model value	Parameter	Model value
V_{bGLYc}		V_{cGLYm}	
K_m	150	$K_{m,cgly}$	5700
V_{max}	4600	V_{max}	10000
k_{out}	1	$K_{m,mgly}$	5700
V_{max}	10000/3		
V_{bSERc}		V_{cSERm}	
K_m	150	$K_{m,cser}$	5700
$Vmax$	2700	V_{max}	10000
k_{out}	1	$K_{m,mser}$	5700
V_{max}	10000/3		
V_{bMETc}	$V_{mHCOOHc}$		
K_m	150	k_{in}	100/3
V_{max}	913	k_{out}	100
k_{out}	1		
V_{bCYSc}	V_{bGlutc}		
K_m	2100 K_m	300	

Table 7 GSH and GSSG transport parameters

Parameter	Model value	Parameter	Model value
V_{cgshHb}	V_{cgshLb}		
K_m	150	K_m	3000
V_{max}	150	V_{max}	1100
Hill	3		
V_{cgsgHb}	V_{cgsgLb}		
K_m	1250	K$_m$	7100
V_{max}	40	V_{max}	4025

Table 8 Quinone redox metabolism parameters

Parameter	Model value	Unit
k_{p1}	3600	hr^{-1}
k_{m1}	72000	hr^{-1}
k_{p2}	0.11	$\mu M^{-1}hr^{-1}$
k_{m2}	0.36	$\mu M^{-1}hr^{-1}$
k_{p3}	1.44	$\mu M^{-1}hr^{-1}$
k_{p4}	72000	hr^{-1}
k_{m4}	27000	hr^{-1}
k_{O2}	293.80	μM

Table 9 Glutathione adduct formation and superoxide dismutase parameters

Parameter	Model value	Unit	Ref
k_{QGSH}	0.0036	$\mu M^{-1}hr^{-1}$	[2]
k_{SOD}	144	$\mu M^{-1}hr^{-1}$	[2]

$$\frac{d}{dt}\,{}_m2cf = V_{mSHMT} + V_{mNE} + V_{mGDC} - V_{mMTD} + V_{SDH} + V_{DMGD} \quad (22)$$

$$\frac{d}{dt}\,{}_m1cf = V_{mMTD} - V_{mMTCH} \quad (23)$$

$$\frac{d}{dt}\,{}_m10f = V_{mFTS} + V_{mMTCH} - V_{mFTD} \quad (24)$$

$$\frac{d}{dt}\,{}_cthf = V_{mMS} + V_{DHFR} + V_{cFTD} - V_{PGT} - V_{cFTS} - V_{cSHMT} + V_{cNE} \quad (25)$$

$$\frac{d}{dt}\,{}_c2cf = V_{cSHMT} + V_{cNE} - V_{TS} - V_{MTHFR} - V_{cMTD} \quad (26)$$

$$\frac{d}{dt}\,{}_c1cf = V_{cMTD} - V_{cMTCH} + V_{cFTD} - V_{PGT} - V_{cFTS} - V_{cSHMT} + V_{cNE} \quad (27)$$

$$\frac{d}{dt}\ {}_c10f = V_{cFTS} + V_{cMTCH} - V_{cFTD} - V_{ART} - V_{PGT} \tag{28}$$

$$\frac{d}{dt}\ dhf = V_{TS} - V_{DHFR} \tag{29}$$

$$\frac{d}{dt}\ 5mf = V_{MTHFR} - V_{MS} \tag{30}$$

$$\frac{d}{dt}\ aic = V_{PGT} - V_{ART} \tag{31}$$

$$\frac{d}{dt}\ met = V_{BHMT} + V_{MS} + V_{cMetc} - V_{MATI} - V_{MATIII} \tag{32}$$

$$\frac{d}{dt}\ sam = V_{MATI} + V_{MATIII} - V_{GNMT} - V_{DNMT} - \tag{33}$$

$$\frac{d}{dt}\ sah = V_{GNMT} + V_{DNMT} - V_{SAHH} \tag{34}$$

$$\frac{d}{dt}\ hcy = V_{SAHH} - V_{CBS} - V_{BHMT} - V_{MS} \tag{35}$$

$$\frac{d}{dt}\ {}_mgly = V_{mSHMT} - 3V_{mGLYc} - V_{GDC} + V_{SDH} \tag{36}$$

$$\frac{d}{dt}\ {}_mser = -3V_{mSERc} - V_{mSHMT} \tag{37}$$

$$\frac{d}{dt}\ {}_mcoo = -3V_{mHCOOHc} - V_{mFTS} \tag{38}$$

$$\frac{d}{dt}\ {}_cgly = V_{bGLYc} + V_{cSHMT} + V_{mGLYc} - V_{GNMT} - V_{GS} \tag{39}$$

$$\frac{d}{dt}\ {}_cser = V_{bSERc} + V_{mSERc} - V_{cSHMT} - V_{CBS} - (1.2) \tag{40}$$

$$\frac{d}{dt}\ {}_ccoo = V_{mHCOOHc} - V_{cFTS} \tag{41}$$

$$\frac{d}{dt}\ dmg = V_{BHMT} - V_{DMDG} \tag{42}$$

$$\frac{d}{dt}\ {}_src = V_{GNMT} + V_{DMGD} - V_{SDH} \tag{43}$$

$$\frac{d}{dt}\ {}_cyt = V_{CBS} - V_{CTGL} \tag{44}$$

$$\frac{d}{dt}\ {}_ccys = V_{CTGL} - V_{GCS} + V_{bCYSc} - (.35)\frac{[{}_ccys]^2}{200} \tag{45}$$

$$\frac{d}{dt}\ {}_cglu = V_{bGLUc} - V_{GCS} - (0.7)glu \tag{46}$$

$$\frac{d}{dt} glc = V_{GCS} - V_{GS} \tag{47}$$

$$\frac{d}{dt}) \, {}_c gsh = V_{GS} - V_{cgshHb} - V_{cgshLb} - 2V_{GPX} + 2V_{GR} - (.002)_c gsh - R_5 \tag{48}$$

$$\frac{d}{dt} \, {}_c gsg = V_{GPX} - V_{GR} - V_{cgsgHb} - V_{cgshLb} - (.1)_c gsg \tag{49}$$

$$\frac{d}{dt} \, {}_b gly = (90)_b gsh + (2)(67.5)_b gsg + V_{oGLYb} - V_{bGLYc} - (.1)V_{bgly} \tag{50}$$

$$\frac{d}{dt} \, {}_b cys = (90)_b gsh + (2)(67.5)_b gsg + V_{oCYSb} - V_{bCYSc} - (.35)V_{bcys} \tag{51}$$

$$\frac{d}{dt} \, {}_b gsh = V_{cgshHbGPX} + V_{cgsgLb} - (.7)_b gsh - (90)_b gsh \tag{52}$$

$$\frac{d}{dt} \, {}_b gsg = V_{cgsgHb} + V_{cgshLb} - (7.5)_b gsg - (67.5)_b gsg \tag{53}$$

$$\frac{d}{dt} \, {}_b glu = (90)_b gsh + (2)(67.5)_b gsg - V_{oGLUb} - (.1)_b glu \tag{54}$$

$$\frac{d}{dt} Q = -R_1 + R_2 - R_5 \tag{55}$$

$$\frac{d}{dt} SQ^{\cdot -} = +R_1 - R_2 - R_4 \tag{56}$$

$$\frac{d}{dt} H_2Q = +R_4 \tag{57}$$

$$\frac{d}{dt} O_2^{\cdot -} = +R_2 - 2R_3 - 2R_6 \tag{58}$$

$$\frac{d}{dt} \, {}_c H_2O_2 = +R_3 - V_{GPX} + R_6 \tag{59}$$

Appendix D: Model validation figures

The combined model was validated by recapitulating figures 2 and 6 from the Reed et. al glutathione metabolism model manuscript [1], in order to confirm that first, addition of quinone redox metabolism does not alter glutathione metabolism when no quinone is present, and second, that the curated model downloaded from the BioModels database was in good accordance with the original published model.

D.1 Reed. et. al. glutathione metabolism Figure 2: GSH half-life

D.2 Reed. et. al. glutathione metabolism Figure 6: effects of oxidative stress

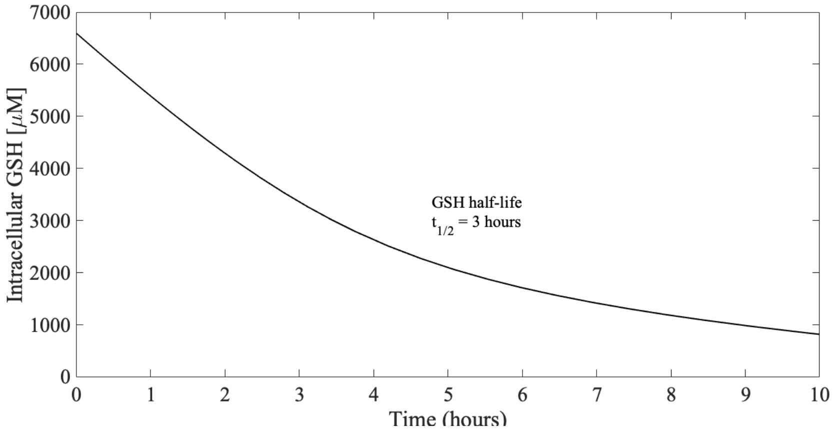

Figure 1 GSH half-life. recapitulation of the Reed et. al. simulations of GSH half-life using the combined model.

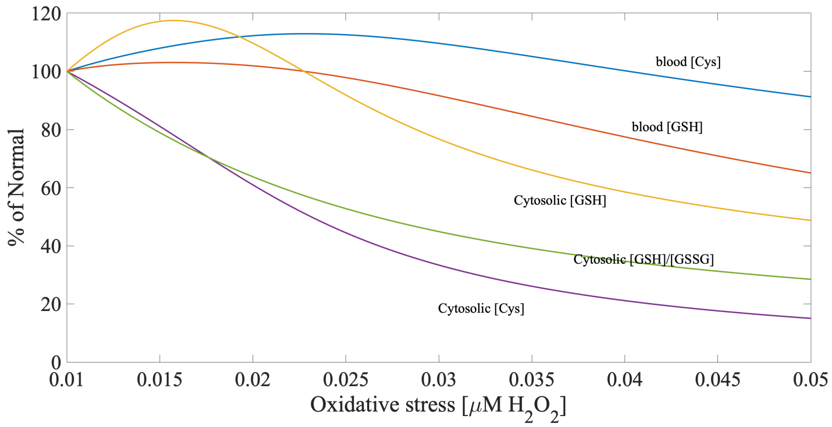

Figure 2 Effects of oxidative stress. recapitulation of the Reed et. al. simulations of oxidative stress using the combined model.

Appendix E: Reduced and comproportionation model fits

The goodness of fit between the OCR data and the reduced and comproportionation models are illustrated in this section. The reduced model falls significantly short of delivering an accurate representation of the OCR data, suggesting that it is mechanistically insufficient to simulate auto-oxidation of doxorubicin. The comproportionation model however, provides a good fit, similar to that of the triad model. However, as described in the manuscript, the triad model is chosen using the AIC-BIC criterion based on the fewer amount of parameters.

E.1 Reduced model fit

E.2 Comproportionation model fit

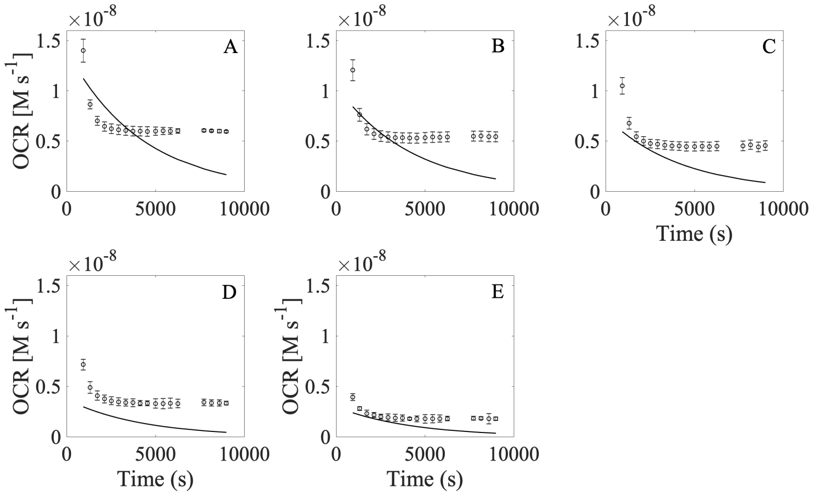

Figure 3 Reduced model fitting analysis recapitulation of OCR data using the reduced model.

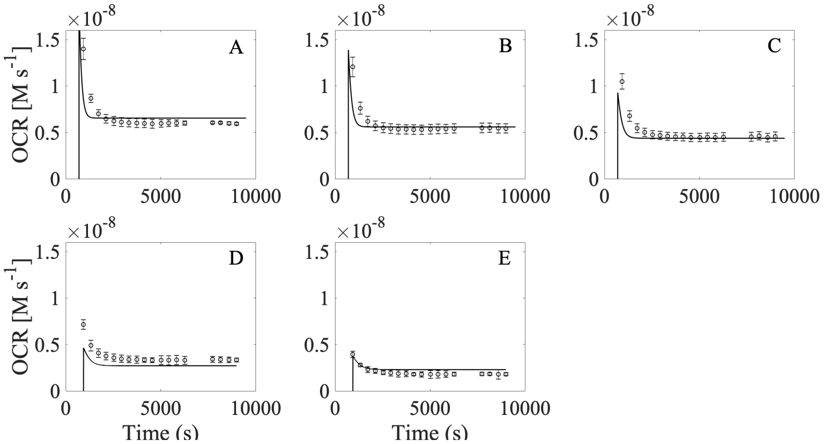

Figure 4 Comproportionation model fitting analysis recapitulation of OCR data using the comproportionation model.

Appendix F: Initial conditions

Initial concentrations for all model variables are split into two sections: 1) initial conditions for the quinone redox metabolism and 2) initial conditions for the glutathione metabolism.Quinone redox metabolism initial conditions for the 50 μM are described in Table 10:

Table 10 Quinone redox metabolism initial conditions

Variable	Conc $[\mu M]$
$[Q]$	50
$[SQ^{\cdot-}]$	0
$[H_2Q]$	0
$[O_2^{\cdot-}]$	0
$[H_2O_2]$	0.01

The remaining initial conditions for all glutathione metabolism variables can be found on the BioModels Database: www.ebi.ac.uk/biomodels-main/BIOMD0000000268

Author details

[1]Department of Applied Mathematics, Liverpool John Moores University, Byrom Street, Liverpool, UK. [2]EPSRC Liverpool Centre for Mathematics in Healthcare, Department of Mathematical Sciences, University of Liverpool, Liverpool, UK. [3]Institute for Risk and Uncertainty, University of Liverpool, Liverpool, UK. [4]School of Pharmacy and Biomolecular Sciences, Liverpool John Moores University, Liverpool, UK. [5]GlaxoSmithKline, David Jack Centre for Research, Ware, UK. [6]MRC Centre for Drug Safety Science, Department of Molecular and Clinical Pharmacology, University of Liverpool, Liverpool, UK.

References

1. Reed, M.E., Thomas, R.L., J, P., J, J.S., Ulrich, C.M., Nijhout, H.F.: A mathematical model of glutathione metabolism. Theor. Biol. Med. Model. **8**, 919–962 (2008)
2. Song, Y., Buettner, G.R.: Thermodynamic and kinetic considerations for the reaction of semiquinone radicals to form superoxide and hydrogen peroxide. Free Radic. Biol. Med. **49** (2010)
